# Repellency and insecticidal activity of seven Mugwort (*Artemisia argyi*) essential oils against the malaria vector *Anopheles sinensis*

**DOI:** 10.1038/s41598-022-09190-0

**Published:** 2022-03-29

**Authors:** De-Yue Luo, Zhen-Tian Yan, Lin-Rong Che, Junwei Jerry Zhu, Bin Chen

**Affiliations:** 1 Chongqing Key Laboratory of Vector Insects, Institute of Entomology and Molecular Biology, Chongqing Normal University, Chongqing, 401331 China; 2 USDA-ARS, AMRU, Lincoln, NE 68583 USA

**Keywords:** Entomology, Natural products, Chemical biology

## Abstract

*Anopheles sinensis* is the main vector of malaria with a wide distribution in China and its adjacent countries. The smoke from burning dried mugwort leaves has been commonly used to repel and kill mosquito adults especially in southern Chinese provinces. In this study, the essential oils of mugwort leaves collected from seven provinces in China were extracted by steam distillation and their chemical compositions were analyzed. Among a total of 56–87 chemical constituents confirmed by GC–MS analyses, four compounds, eucalyptol, β-caryophyllene, phytol and caryophyllene oxide, were identified with appearances from all seven distilled essential oils. The effectiveness varied in larvicidal, fumigant and repellent activities against *An*. *sinensis* from these seven essential oils with different geographic origins. The essential oil from Hubei province showed the highest larvicidal activity against the 4th instar larvae of *An. sinensis*, with a median lethal concentration at 40.23 µg/mL. For fumigation toxicity, essential oils from 4 provinces (Gansu, Shandong, Sichuan and Henan) were observed with less than 10 min in knockdown time. The essential oil distilled from Gansu province displayed the highest repellent activity against *Anopheles* mosquitoes and provided similar level of protection as observed from DEET. Eucalyptol was the most toxic fumigant compound and phytol showed the strongest larvicidal activity among all tested mugwort essential oil constituents.

## Introduction

Mosquitoes are considered as important medical insect pests worldwide, and they, as vectors, transmit many diseases including malaria, filariasis, yellow fever and dengue fever^1–3^. Malaria has been reported as the most common mosquito-borne disease and present a great threat to human life mainly spread by *Anopheles* mosquitoes^4^. Every year, approximately 250 million people have been reported being infected with malaria, which has caused over one million deaths worldwide^5^. *Anopheles sinensis* is the No.1 vector of malaria in China^6^. The control of this vector is mainly managed with massive applications of various insecticides. However, the over uses of these toxic chemicals have resulted in strong insecticide resistance^7,8^.

Currently for mosquito control in China, pyrethroids are primary chemical insecticides being heavily used. N,N-Diethyl-3-methylbenzamide (DEET) is the most commonly repellent used for the prevention against *An. sinensis*. However, extensive use of pyrethroids has caused increasing insecticide resistance, and also created adverse effects on human health and their surroundings^9–11^. The toxicology of DEET has been more closely investigated and has found issues in safety for human use, including use on children, pregnant women, and lactating women^12–14^. There is an urgent need to develop alternative human and environmentally friendly mosquito control strategies. In recent years, natural products have gradually become the primary sources of novel mosquito repellents and larvicides due to low-toxic, quick biodegradation rates, and less side effects on hosts and non-target organisms^15–17^. Plant essential oils, extracted from *Nepeta cataria*, *Severinia monophylla*, *Syzygium aromaticum* and *Cymbopogon citratus* have been reported to be effective in repelling mosquito adults^18–20^. Besides, some of them have also exhibited larvicidal activity, including those from *Laurencia dendroidea*, *Cunninghamia konishii* and *Azadirachta indica*^21–23^.

The Chinese mugwort plant, *Artemisia argyi*, is a traditional medicine plant widely distributed in southern provinces with a long cultivation history. Extracts of this plant have been reported to possess immunomodulatory, neuroprotective insecticidal properties with effectiveness against bacteria, dampness, hemostasis, cancer, and inflammation^24–26^. Mugwort plants have been widely used to repel mosquitoes by burning the harvested dried leaves for thousands of years at countryside in China^27^. The essential oil of those plant leaves exhibits strong contact fumigant toxicity against *Lasioderam serricorne* adults^28^. The oils of four subspecies of mugwort plants including *Ar. feddei*, *Ar. gmelinii*, *Ar. manshurica*, and *Ar. olgensis* deterred mosquito biting of adult *Ae. aegypti*^29^. The essential oil of *Ar. vulgaris* provided 100% mortality against *Ae. aegypti* larvae after 8-h exposure at a concentration of 500 ppm.

In the study, we extracted the essential oils from plants of *Ar. argyi* collected from seven provinces/municipal areas using a steam distillation technique, and investigated their larvicidal, fumigant and repellent activity against *An. sinensis*. We also analyzed their chemical compositions using gas chromatography mass spectrometer (GC–MS), and evaluated the toxicity of four common predominant compounds of these oils against *An. sinensis*. The results from this study may provide some new insights for further research in use of the mugwort essential oil as an alternative and targeted control and preventative measure against *Anopheles* mosquitoes.

## Results

### Insecticidal and repellent activity of distilled *Ar. argyi* essential oils

The essential oil of Hubei province (HB) showed the highest larvicidal effect with a median lethal concentration (LC_50_) value at 40 μg/mL, followed by oils from mugwort plants collected in Chongqing (CQ) (LC_50_ = 49 μg/mL), Sichuan (SC) (LC_50_ = 54 μg/mL), Hainan (HN) (LC_50_ = 56 μg/mL), Gansu (GS) (LC_50_ = 55 μg/mL) and Shandong (SD) (LC_50_ = 59 μg/mL) and Yunnan (YN) (LC_50_ = 62 μg/mL) (Table 1). The larvicidal activity of all seven essential oils are significantly effective against *An. sinensis* in comparison of control in which no dead larvae were observed.

**Table 1:** Larvicidal activity of essential oils distilled from seven geographical regions of mugwort leaves in China against the 4th instar larvae of *An. sinensis*.

Oil^a^	LC_50_ (μg/mL) ± SE^b^	95% Confidence limits	Toxicity regression equation (y = a + bx)^c^	χ2
CQ	48.77 ± 7.40	41.60–55.18	y = -1.85 + 0.03x	3.21*
SC	54.24 ± 6.30	49.12–59.41	y = -3.14 + 0.05x	2.18*
YN	61.82 ± 5.48	53.61–69.39	y = -1.77 + 0.03x	4.51*
HB	40.23 ± 1.0	32.42–46.72	y = -1.59 + 0.04x	4.95*
HN	55.93 ± 0.29	49.51–62.12	y = -2.24 + 0.04x	2.99*
SD	59.31 ± 6.40	51.27–66.70	y = -1.76 + 0.03x	0.37*
GS	55.20 ± 8.50	48.00–61.88	y = -1.92 + 0.03x	2.58*

^a^CQ, SC, YN, HB, HN, SD and GS: essential oils from Chongqing, Sichuan, Yunnan, Hubei, Henan, Shandong and Gansu province/municipality, respectively. ^b^Determined by log-probit analysis with at least 6 concentrations, and three repetitions “SE” denote standard error. ^c^y stands for the probability transformation value of percent mortality of mosquitoes, and x for the concentration of essential oil, respectively. *Significant at *p* < 0.05.

**Figure 1:**
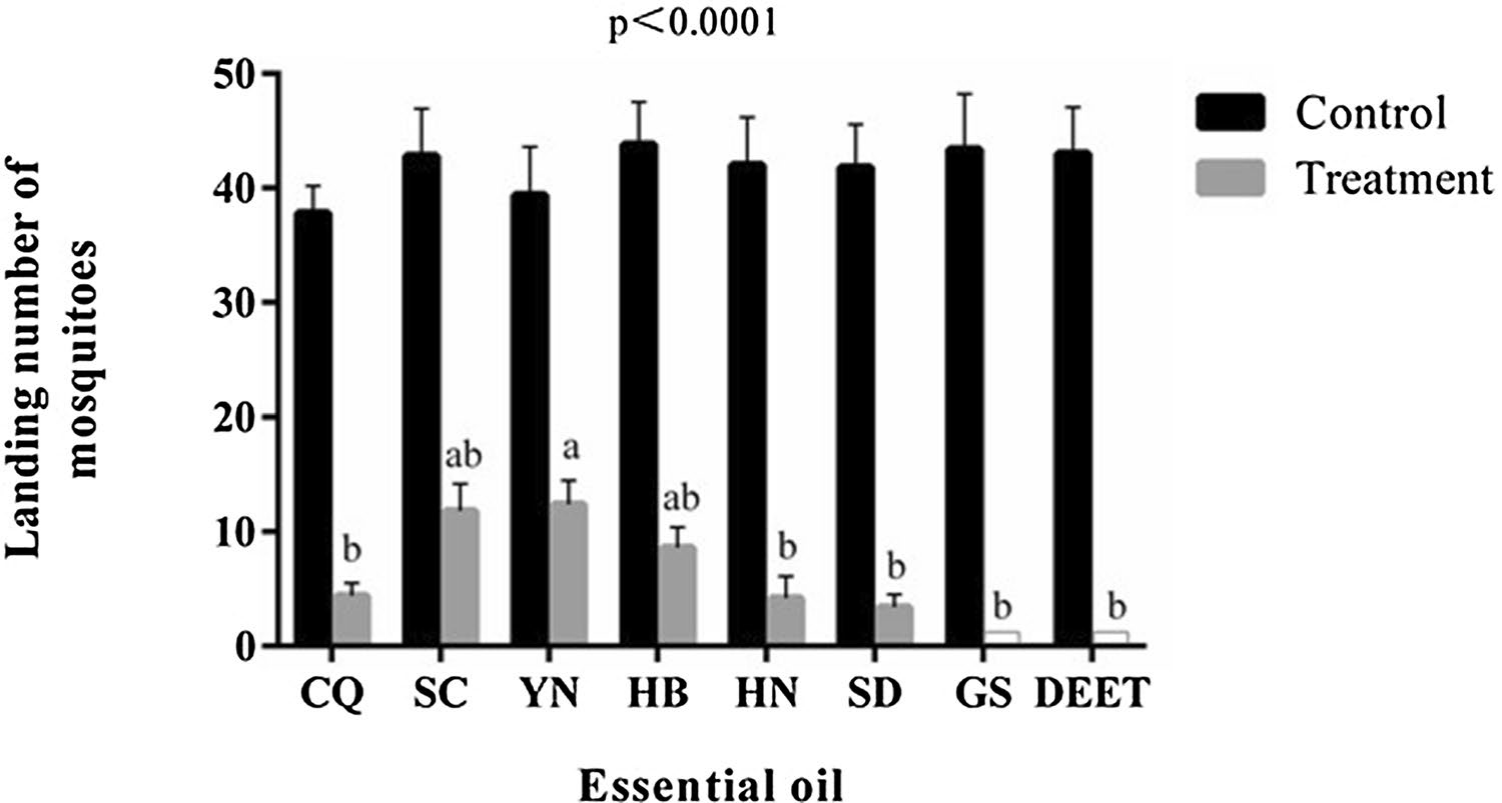
The first 5-min observed landing rates of starved *An. sinensis* on human skins treated with seven *Ar. argyi* essential oils extracted from different geographical areas. The significances between each treatment and control group are less than 0.0001 (p < 0.0001). Different letters on each treatment bars indicate significantly different between among the seven essential oils tested and the positive control, DEET (ANOVA).

All seven oils showed significant repellent activities against female *An. sinensis* adults compared to the negative control (Fig. 1). Similar levels of repellency as DEET were demonstrated from 4 oils (CQ, HN, SD and GS), with the highest from GS oil. The mean % of repellent longevity from seven mugwort essential oils were presented in Table 2. The essential oil extracted from leaves collected in GS provided almost 66% of protection against mosquitoes at the 65th minutes post application, which was similar to the effectiveness found from 10% DEET.

**Table 2:** The percentages of repellencies of seven *Ar. argyi* essential oils and DEET against the four-day old *An. sinensis* adults during different exposed times*.*

Oil treatments (1.5 µL/cm^2^)^a^	Minutes post application*				
	5 min	20 min	35 min	50 min	65 min
CQ	88.32 ± 3.19^b^	79.18 ± 1.63^b^	66.82 ± 3.20^b^	57.49 ± 5.85^b^	34.55 ± 5.02^b^
SC	72.53 ± 3.95^e^	61.41 ± 1.33^c^	42.81 ± 2.37^d^	27.44 ± 1.38^d^	14.78 ± 1.05^c^
YN	68.62 ± 2.93^e^	58.91 ± 1.15^c^	37.56 ± 0.67^d^	22.30 ± 1.58^d^	9.02 ± 2.33^c^
HB	80.09 ± 3.03^c^	74.98 ± 1.74^b^	57.97 ± 5.65^c^	40.04 ± 1.73^c^	28.94 ± 2.68^b^
HN	90.21 ± 3.62^b^	80.51 ± 2.02^b^	69.30 ± 4.40^b^	59.20 ± 2.51^b^	37.29 ± 2.34^b^
SD	91.90 ± 2.42^b^	81.48 ± 2.47^b^	71.67 ± 5.95^b^	61.31 ± 1.49^b^	39.43 ± 2.94^b^
GS	100 ± 0.00^a^	99.57 ± 0.97^a^	94.28 ± 3.21^a^	76.01 ± 1.06^a^	65.68 ± 3.26^a^
DEET (10%)	100 ± 0.00^a^	99.59 ± 0.91^a^	95.04 ± 3.21^a^	83.27 ± 3.69^a^	79.81 ± 4.28^a^

*The repellent rates with different superscript letters in the same column are significantly different at *p* < 0.05. The rates were determined with three replications. CQ, SC, YN, HB, HN, SD and GS: essential oils from Chongqing, Sichuan, Yunnan, Hubei, Henan, Shandong and Gansu province/municipality, respectively.

### Fumigant toxicity of distilled *Ar. argyi* essential oils

The median knockdown time (KT_50_) ranged from as short as 4.7 min from essential oils from GS, to over 50 min of oils from YN province (Table 3). Similar trends appeared also in the median lethal dose (LD_50_), which oils of GS showed the strongest toxicity against the female adults of *An. sinensis*, and the least from oils of YN. The essential oil from GS has the highest fumigant toxicity with a LD_50_ value at 9.40 μL/L (Table 4).

**Table 3:** Median lethal knockdown activity (mins) of the essential oils extracted from seven geographical regions of *Ar. argyi* against female adults of *An. sinensis*.

Oil	KT_50_ (min)^a^	95% Confidence limits	Toxicity regression equation (y = a + bx)	**χ**2
CQ	13.85 ± 3.5	13.08–14.62	y = −4.33 + 0.31x	0.29*
SC	10.18 ± 3.4	9.43–10.91	y = −3.71 + 0.36x	0.83*
YN	50.62 ± 8.6	48.19–53.52	y = −2.69 + 0.05x	12.95*
HB	16.77 ± 4.7	15.39–18.08	y = −1.87 + 0.11x	5.55*
HN	10.02 ± 2.8	9.02–10.96	y = −2.21 + 0.22x	3.02*
SD	9.32 ± 2.2	8.61–10.05	y = −3.36 + 0.36x	2.84*
GS	4.71 ± 1.2	3.50–5.32	y = −2.45 + 0.51x	0.33*

^a^KT_50_ represents the knockdown time of half individuals, which was determined by log-probit analysis. Three experimental repeats with the essential oil dosage of 2 µL. *Significant at *p* < 0.05. CQ, SC, YN, HB, HN, SD and GS: essential oils from Chongqing, Sichuan, Yunnan, Hubei, Henan, Shandong and Gansu province/municipality, respectively.

**Table 4:** Fumigation toxicity of the essential oils extracted from seven geographical regions of *Ar. argyi* in China against female adults of *An. sinensis*.

Oil	LD_50_ (μL/L)^a^	95% confidence limit	Toxicity regression equation (y = a + bx)	**χ**2
CQ	14.95 ± 7.8	13.32–16.43	y = −1.65 + 0.11x	2.15*
SC	22.90 ± 5.5	20.82–25.08	y = −1.56 + 0.06x	2.91*
YN	51.26 ± 6.1	42.87–71.69	y = −2.36 + 0.04x	2.77*
HB	48.74 ± 2.1	39.09–76.29	y = −1.39 + 0.02x	4.15*
HN	26.49 ± 4.5	24.25–29.13	y = −1.70 + 0.06x	3.00*
SD	18.66 ± 1.2	16.75–20.44	y = −1.54 + 0.08x	3.55*
GS	9.40 ± 3.6	5.15–12.08	y = −1.51 + 0.16x	8.62*

^a^Determined with at least 6 concentrations at μL/L. *Significant at *p* < 0.05. CQ, SC, YN, HB, HN, SD and GS: essential oils from Chongqing, Sichuan, Yunnan, Hubei, Henan, Shandong and Gansu province/municipality, respectively.

### Chemical compositions of *Artemisia argyi* essential oils

In total, 56–87 chemical constituents were identified from *Ar. argyi* essential oils from seven geographical origins, with the highest number of compounds (87) found in the oil from Gansu province (Table 5). Four chemical compounds, β-caryophyllene, caryophyllene oxide, eucalyptol and phytol were the most abundant and detected from all seven oils. There were significant differences in compositions of various compounds among oils extracted from seven different regions. Eucalyptol was found over 20% in GS essential oils, but only 5.5% from YN province. Only one oil from HB province contained over 16% phytol, with less than 10% from oils of the rest of regions. Another monoterpenoid, thujone, was detected over 16% from the SC oil.

**Table 5:** GC–MS analyses of compositional compounds of the essential oils extracted from seven regions of *Ar. argyi*.

Constituents	CAS^a^	RI–A^b^	RI–B^c^	Constituent percentage (%)						
				CQ	SC	YN	HB	HN	SD	GS
Santolina triene	2153-66-4	908	907	–	–	12.36	–	–	–	–
1R-α-Pinene	7785-70-8	929	932	2.58	1.38	2.49	–	–	1.47	–
α-Pinene	80-56-8	937	932	–	–	–	–	–	–	3.32
Camphene	79-92-5	952	947	–	1.24	–	–	1.19	1.02	2.13
Sabinen	3387-41-5	974	973	–	1.81	4.48	–	–	–	1.28
β-Pinene	127-91-3	979	977	1.62	2.02	–	–	1.08	–	2.26
( +)-2-Carene	149,946	991	1015	–	–	–	–	–	1.16	–
Yomogi alchool	26,127-98-0	1000	1000	–	–	–	–	1.47	1.23	1.32
α-Phellandrene	99-83-2	1005	1003	–	–	1.91	–	–	–	–
o-Cymene	527-84-4	1022	1022	1.74	1.78	4.39	1.18	–	–	1.58
m-Cymene	535-77-3	1023	1022	–	–	–	–	1.68	–	–
p-Cymene	99-87-6	1025	1023	–	–	–	–	–	1.76	–
**Eucalyptol**	470-82-6	1032	1031	**11.44**	**12.62**	**5.49**	**15.67**	**8.68**	**4.86**	**21.89**
Artemisia ketone	546-49-6	1062	1061	–	–	–	–	–	2.02	2.76
cis-Sabinene hydroxide	17,699-16-0	1070	1068	–	–	–	–	1.19	4.33	3.93
Artemisia alcohol	27,644-04-8	1084	1084	2.26	–	–	1.34	4.81	2.82	3.55
Thujone	546-80-5	1103	1107	–	16.66	–	–	4.18	1.31	–
Isothujone	471-15-8	1114	1117	–	–	–	–	1.22	–	–
Chrysanthone	1125-12-8	1119	1108	–	–	–	4.97	–	–	–
Isochrysanthenone	473-06-3	1123	1125	4.08	1.8	–	–	–	–	1.84
(-)-Camphor	464-48-2	1142	1143	–	1.49	2.15	–	3.24	2.12	5.58
( +)-2-Bornanone	471-16-9	1143	1143	–		–	3.05	–	2.21	–
p-Menth-8-en-1-ol, cis	7299-41-4	1144	1066	–	1.35	–	–	–	–	–
Isoborneol	124-76-5	1157	1166	–	1.41	–	–	2.55	2.62	–
endo-Borneol	507-70-0	1167	1167	–	–	–	6.22	–	1.85	3.37
β-Artemisia acetate	3465-88-1	1173	1174	1.51	–	–	–	1.43	1.03	1.4
Terpinen-4-ol	562-74-3	1177	1178	–	–	–	–	1.74	–	–
L-4-terpineol;	20,126-76-5	1182	1181	–	1.25	–	1.6	–	–	1.29
α-Terpineol	98-55-5	1189	1192	–	–	–	–	–	–	1.98
S-Verbenone	1196-01-6	1204	1397	1.78	–	–	–	–	–	–
Piperitol isomer	16,721-39-4	1208	1210	–	–	–	–	–	1.29	–
cis-Mentha-1,8-dien-6-ol	1197-06-4	1229	1221	–	–	–	1.66	–	1.18	–
2,3-Pinanediol	53,404-49-2	1244	1317	–		1.03	–	–	–	–
Neryl acetate	141-12-8	1364	1292	–	–	2	–	–	–	–
Ylangene	14,912-44-8	1372	1375	–	–	1.83	–	–	–	–
Copaene	3856-25-5	1376	1376	1.11	–	–	–	–	–	–
(-)-β-Bourbonene	5208-59-3	1384	1384	1.06	–	–	–	–	–	–
1H-Cyclopropazulene	489-40-7	1409	1409	–	1.03	–	–	–	–	–
**β-Caryophyllene**	87-44-5	1419	1419	**14.45**	**12.25**	**8.87**	**7.62**	**11.45**	**15.54**	**3.17**
Humulene	6753-98-6	1454	1454	1.77	1.96	2.78	1.47	1.87	1.04	–
(E)-β-Famesene	18,794-84-8	1457	1459	2.85	1.51	1.17	–	2.2	–	–
Germacrene D	23,986-74-5	1481	1481	7.61	–	8.72	4.85	6.93	4.86	4.75
β-Selinene	17,066-67-0	1486	1486	2.54	–	2.75	1.15	–	1.06	1.43
( +)-Bicyclogermacrene	24,703-35-3	1495	1497	–	1.97	1.51	–	1.14	–	–
**Caryophyllene oxide**	1139-30-6	1581	1584	**13.24**	**10.64**	**8.56**	**12.51**	**14.82**	**9.45**	**9.63**
Neointermedeol	5945-72-2	1660	1660	–	2.84	–	5.13	3	4.72	4.91
11,11-Dimethyl-4,8-dimethylenebicycloundecan-3-ol	79,580-01-1	1646	1640	–	–	–	1.18	–	1.01	–
Hexahydrofarnesyl acetone	502-69-2	1844	1847	1.25	–	–	1.64	1.49	1.2	1.45
n-Hexadecanoic acid	57-10-3	1968	1984	–	–	–	–	1	–	–
pentenylcurcumene	55,968-43-9	1980	2121	–	–	1.93	–	–	–	–
**Phytol**	150-86-7	2114	2115	**8.29**	**7.02**	**6.3**	**16.28**	**9.95**	**8.27**	**8.02**
Elemene isomer	–	1344	1497	1.18	–	–	–	–	–	–
Terpineol	8006-39-1	–	–	–	–	–	1.43	1.05	2.27	–
**Total %**				**82.36**	**84.03**	**80.72**	**88.95**	**89.36**	**83.7**	**92.84**

^a^CAS: Chemical Abstracts Service Registry Number. ^b^Retention index from NIST references (qualitative index of gas chromatography). ^c^Retention index calculated with N alkanes (C_5_–C_25_Hc) as standard. The chemical constituents with less than 1% composition in each essential oil are not listed in the table. CQ, SC, YN, HB, HN, SD and GS: essential oils from Chongqing, Sichuan, Yunnan, Hubei, Henan, Shandong and Gansu province/municipality, respectively.

### Larvicidal and fumigant toxicity of the 4 dominant compositional compounds

All four individual compounds showed significant toxicity against both larvae and adults of *An. sinensis* (Tables 6 and 7). Phytol was the strongest larvicidal compound, with the LC_50_ at 16.03 μg/mL. Significant larvicidal activity was also found from caryophyllene oxide (LC_50_ = 39.09 μg/mL), relative to those of eucalyptol and β-caryophyllene (Table 6). Eucalyptol exhibited the highest fumigant lethal activity against adult mosquitoes among the four tested compounds with the shortest time to kill at the LT_50_ value of 5.48 μL/L.

**Table 6:** Larvicidal activity of the four individual compounds mostly found from the seven essential oils of *Ar. argyi* in China against the 4th instar larva of *An. sinensis*.

Compounds	LC_50_ (μg/mL)	95% Confidence limits	Toxicity regression equation (y = a + bx)	χ2
eucalyptol	155.55 ± 6.7	127.34–243.40	y = −2.33 + 0.01x	1.58*
β-caryophyllene	134.77 ± 8.9	119.06–171.96	y = −3.27 + 0.02x	1.11*
caryophyllene oxide	39.09 ± 5.2	31.28–45.55	y = −1.55 + 0.04x	1.73*
phytol	16.03 ± 2.6	−12.77–30.76	y = −0.32 + 0.02x	1.79*

*Significant at *p* < 0.05.

**Table 7:** Fumigation toxicity of the four individual compounds mostly found from the seven essential oils of *Ar. argyi* in China against the female adults of *An. sinensis*.

Compounds	LT_50_ (μL/L)	95% Confidence limits	Toxicity regression equation (y = a + bx)	χ2
Eucalyptol	5.48 ± 3.4	3.50–6.65	y = −1.37 + 0.25x	0.05*
β-caryophyllene	32.60 ± 4.1	30.36–35.62	y = −2.58 + 0.07x	3.78*
Caryophyllene oxide	26.02 ± 5.3	24.60–29.16	y = −2.03 + 0.03x	3.26*
Phytol	17.98 ± 2.2	15.49–20.21	y = −1.13 + 0.06x	3.45*

*Significant at *p* < 0.05 level.

## Discussion

It is not surprising that the essential oils of the mugwort plant leaves have shown strong larvicidal activity against *An. sinensis*, as Wang^30^ have previously reported that *Ar. argyi* essential oil exhibiting larvicidal activity against *Culex* mosquitoes. Interestingly, significant differences in larvicidal activity have been demonstrated among the *Ar. argyi* oils from 7 different geographic regions, which may result from various quantities of some terpene and monoterpenoid compounds as their major compositional constituents^31^. Eucalyptol, β-caryophyllene, caryophyllene oxide and phytol, the four most common compounds found from all distilled oils in seven geographic origins, have been reported with moderate to strong larvicidal activity against various mosquito larvae. For instance, phytol has been reported with larvicidal activity against *Aedes* and *Culex* mosquitoes as one of compositional compounds from several essential oils^32–35^. From the distilled mugwort leaf essential oils, phytol is also one of the most dominant compositional compounds (6.3–16.3%). Eucalyptol is another most abundant compound ranged from 5–22% among all identified constituents. The essential oil from HB displays the highest larvicidal activity with over 52% of eucalyptol and phytol presence. Eucalyptol also shows larvicidal activity against larvae of *An. anthropophagous*, LC_50_ values from 45–50 μg/mL^36^. However, eucalyptol has displayed a relatively weaker larvicidal activity at LC_50_ of 155 ug/mL, which is one third of what reported from above mentioned studies and ten times less than that of phytol (LC_50_ = 16 μg/mL) in the present study. Interestingly, a relatively strong activity of caryophyllene has been demonstrated (LC_50_ = 39 μg/mL). Similar findings of weak larvicidal activity from eucalyptol identified from oil of *Ar. gilyescens* have been reported against *An. sinesis* with LC_50_ > 200 μg/mL^37^. Although we have shown that β-caryophyllene is a much weaker larvicide than that of caryophyllene oxide against *An. sinesis* in the present study, Govindarajan et al.^38^ found that β-caryophyllene from *Plectranthus barbatus* essential oil performed a strong larvicidal activity against *Aedes* and *Culex* mosquito larvae, and larvae of *An. subpictus*. Furthermore, Zhu and Tian^37^ have reported a strong larvicidal activity against *An. anthropophagus*, with only 5% of caryophyllene oxide constituently from the essential oil of *Ar. gilvescene*. Obviously, synergistic effects may play additional roles to enhance the larvicidal activity from various essential oil constituent compounds.

The moxibustion therapy of using *Ar. argyi* has been considered as a form of complementary or alternative medicine existing in the holistic system of health care and healing in many parts of Southeast Asia^39^. Burning of *Artemisia* dried leaves has been widely used to repel mosquitoes by minority people living in remote areas in southern provinces of China^40^. Among the four most dominant constituent compounds, eucalyptol has been reported acting as a strong repellent against various species of adult mosquitoes^41^. In the present study, the essential oil extracted from Gansu province has demonstrated a high level of protection as found from DEET, which could be contributed by containing over 22% of eucalyptol among other compositional compounds. Eucalyptol, also named 1,8-cineole, has been reported as a strong mosquito repellent identified from many species of *Eucalyptus, Ocimum* and *Lippia*^15,31^. Fumigant test with individual compound (eucalyptol) alone has further supported its strongest toxicity against adult *An. sinensis* mosquitoes. Phytol, a linear dipterpene alcohol, has been reported with high repellent activity against *An*. *gambiae* and *Ae. aegypti*^42,43^. β-Caryophyllene identified from essential oils of seven different provinces only provides low level of repellent activity and fumigant toxicity, which similar results have been reported from essential oils of *Nepeta cataria*, *Lippia camara* and *Lippia cheraliera *etc.^44,45^. A detailed literature review by Mathew and Thoppil^32^ has shown that caryophyllene oxide is detected from many essential oils with probable cause for high mosquito larvicidal activity. For instance, caryophyllene oxide is a major constituent compound in essential oils of *Lippia gracilis* and *Hyptis pectinate* with the potent insecticidal activity against *Aedes aegypti* larvae^46^. Strong larvicidal activity was also demonstrated from the essential oil of *Ar. argyi*.

The potential of using essential oils or their constituents as fumigants needs to be explored more thoroughly. In southeastern Asia remote areas and Latin American rural regions, the use of various fumigations for insect repelling and disease vector control has been widely reported^47,48^. Many monoterpenes (eucalyptol), diterpenes (phytol), sesquiterpenes (caryophyllene) as discovered in *Ar. argyi* essential oil have been reported possessing fumigant toxicity^49^. All of these are supported by the strong fumigant toxicity and the shortest knockdown time found from *Ar. argyi* essential oils extracted from Gansu province. Three other species of *Artemisia* essential oils also have the fumigant activity against mosquitoes, including *Ar. scoparia*, *Ar. capillaries* and *Ar. carvifolia*^50^*.*

The chemical composition and content of essential oils of plants are related to the diverse climate^51^. In addition, various elements in the soil also contribute to the differences in the chemical composition of *Ar. argyi* essential oil. For instance, the content of calcium, magnesium, manganese and nickel in Hubei *Ar. argyi* essential oil were higher, the elements of Nickel, cobalt, chromium and zinc in Sichuan *Ar. argyi* essential oil were relatively abundant, and the content of copper and cobalt in Henan *Ar. argyi* essential oil were also higher^52^**.** It was found that phosphorus content in soil affected plant growth, quality and chemical composition content^53^. The content of *Angelica sinensis* volatile oil was 0.65% from Gansu, 0.59% from Yunnan and 0.29% from Sichuan, which were directly related to the longitude and latitude of different regions^54^. In addition to the reasons above, the altitude and water quality may also be another key factor of the chemical composition and content of *Ar. argyi* essential oil^55^, which need further discussion.

This research demonstrates the strong larvicidal activity, fumigant toxicity and repellent effect of *Ar. argyi* essential oils of seven different geographic origins in southern provinces of China against larvae and adult *An. sinensis* mosquitoes. Eucalyptol and phytol are the most abundant constituent compounds and most active compounds among others. Additional minor compounds detected from these oils may enhance insecticidal, repellent and fumigant effects as well, which further studies need to be carried out. Essential oils that constitute an important alternative to conventional insecticides are largely due to their selectivity (high toxicity for mosquitoes but not for other co-existing organisms) and their minimal environmental effects^56^. Essential oils and their constituents, such as monoterpenes, have been widely used as fragrances in cosmetics, food additives, household products and medicine. Many of them are generally recognized as safe by the U.S. Food and Drug Administration (FDA). The outcomes from this study have provided new insights in use of phytochemicals derived from various botanical sources for more targeted mosquito control.

## Materials and methods

### Chemicals

Caryophyllene oxide (> 90%) was obtained from Shanghai Yuanye Technology Co., Ltd. of China. N,N-Diethyl-3-methylbenzamide (DEET, 99%), eucalyptol (99%), β-caryophyllene (> 80%) and phytol (90%) were all purchased from Macklin Biochemical Co., Ltd. (Shanghai, China). The alkanes analytic standards (C_5_–C_25_ Hc,), reagent brand: o2si were purchased from ANPEL Laboratory Technologies (Shanghai, China), with over 99% purity.

### Mosquitoes

*Anopheles sinensis* was originally collected from Jiangsu province in China, and reared in the Institute of Insect and Molecular Biology, Chongqing Normal University, for more than 40 generations. Mosquitoes were reared at a 12:12 light/dark photoperiod, 65 ± 5% relative humidity under 27 ± 1 °C, which larvae were fed with fish food and adults were provided with 10% glucose solution. The 4th instar of larvae and the four-day old adult mosquitoes were used for larvicidal, fumigant and repellent activity assays in this study. All experimental methods of this study are carried out in accordance with relevant guidelines and regulations, and The Ethical Committee of Chongqing Normal University approved the hunan-bait assays performed in the study.

### Plant material collections and essential oil extraction from *Artemisia argyi*

The plant *Ar. argyi* is widely distributed in field in China. All experimental materials of *Ar. argyi* were collected on public lands in field at each location of seven provinces in China from September to October in 2018, and the plant material collecting did not require permission or other relevant certificates. The wet weights of collected aerial branches and leaves were 70 kg from 25 m^2^ of wasteland in Chongqing, 65 kg from 100 m^2^ of wasteland in Sichuan, 46 kg from 200 m^2^ of mountain area in Yunnan, 40 kg from 100 m^2^ of agriculrural farm in Hebei, 78 kg from 35 m^2^ of agricultural field in Henan, 50 kg from 150 m^2^ of mountain area in Shangdong, and 55 kg from 150 m^2^ of mountain area in Gansu, respectively. These seven provinces could represent the main distribution of the *Ar. argyi* plant in China in term of geography and climate. The collected *Ar. argyi* was dried in the collecting locations, and then sent to laboratory at Chongqing Normal University. The samples of *Ar. argyi* plants from each location were identified by the plant taxonomist Prof. Hai He at the Chongqing Normal University where we work, and the specimens are stored in the Herbarium of Chongqing Normal University with following information as attached label: collecting time, collecting location, collector's name, specimen number, species name, and identifier’s name. The dried *Ar. argyi* was cut to pieces at a size of about 10 cm in diameter by guillotine, and the essential oil of *Ar. argyi* was extracted using 6 kg of dried material per location. The extraction lasted for six hours using a steam distillation apparatus, and oil–water mixture was separated by a separatory funnel with an addition of NaCl as described in Joshi et al.^57^. The upper layer from the separated essential oils were taken, and then dried with added anhydrous sodium sulfate^58^. The purified oils were stored in brown reagent bottles at 4 °C prior to bioassays and chemical analysis.

### Larvicidal activity assay

The larvicidal activity of *Ar. argyi* essential oils and their four dominant compounds against *An. sinensis* larvae was tested according to the method introduced by Nyamoita^59^. The range of lethal doses for the investigated samples was determined using the doses at 20, 40, 60, 80, 100 and 120 μg/mL, respectively, and a total of three replicates were conducted. Ethanol was used as the control. Different dosages of samples were added to a 50-mL cup containing a total of 20 mL of dechlorinated water. Twenty 4th instar larvae were then gently moved to the cup and the bioassay was performed under room temperature at 27 ± 1 °C with an 80 ± 10% relative humidity. The number of larvae with no movement (after touching) were considered as dead and were counted after 24 h of exposure.

### Fumigant toxicity assay

The fumigant activity of *Ar. argyi* essential oils and their four dominant compounds against *An. sinensis* adults was investigated using methods described in Jiang et al.^60^, with some changes. Twenty female *An. sinensis* adults at the age of 4-day old were released into a 250 mL of Erlenmeyer flask sealed using a rubber stopper with a predrilled hole (diameter = 0.4 cm) for a 15 min of acclimatization. A piece of filter paper (1 × 3 cm) was then placed into the flask. The filter papers were applied testing materials at doses of 2, 4, 6, 8, 10, 12 μL of essential oils. After one hour of exposures, mosquitoes were then transferred to cages for 24-h provided with cotton balls containing 10% glucose. The number of dead mosquitoes was counted. For the fumigant knockdown test, the dose of testing material was 2 μL^61^, the knockdown number were recorded every five minutes until reaching the first hour. The time of mosquitoes observed falling down at the bottom were considered as the knocked down time, no any movement was recorded as the lethal time. A total of three replicates were performed in each trial. The experiments were carried out at a room temperature of 26 ± 1 °C. Probit analysis was used to calculate LC_50_ and KT_50_ values^62^.

### Repellent activity assay

The repellent assay was carried out using a human-bait technique reported by Kaliyaperumal et al.^63^ with a few modifications. Before the experiment, both hands and forearms of the three volunteers were asked to be washed with scent free soaps, then dried. Gloves wore to cover the entire hand and forearm except for an area of 25 cm^2^ (5 × 5 cm) on the dorsal sides were cut by a pair of scissors. One hundred 24-h starved 3-day-old female *An. sinensis* were released into a screen cage (300 mm × 300 mm × 300 mm) prior to the experiment. About 37.5 µL of testing extracted *Ar. argyi* oils were evenly applied on top of the exposed skin area, then inserted into the testing mosquito cage. 10% DEET repellent formulation was used as positive control. The number of mosquitoes landing on exposed area of skin was counted every fifteen minutes up to the 65th min. Ethanol used as the negative control by applying the same amount onto another hand. Both hands were exposed to starved mosquitoes starting at the same time. Each extracted mugwort oil and 4 individual compounds was repeated three times in random orders. The time intervals of every new volunteers were about 2 h. The percentage of repellency was calculated by using the formula^64^:

$$ {\text{Percentage}}\;{\text{of}}\;{\text{repellency}} = [\left( {{\text{N}}_{{\text{c}}} - {\text{N}}_{{\text{t}}} } \right)/{\text{N}}_{{\text{c}}} \times 100 $$

where N_c_ and N_t_ is the number of mosquito landings on hand of negative control and test samples, respectively. The testing protocol was approved by the Committee of Laboratory Animal Experimentation at Chongqing Normal University (Approval No. Zhao-20200416-01), and all volunteers were provided with the official written informed consent form for testing.

### Chemical analysis of the *Ar. argyi* essential oils

The chemical constituents of *Ar. argyi* essential oils were analyzed using a gas chromatography-mass spectrometer (Thermo Scientific TSQ XLS GC–MS with a model TRACE 1310 GC), which was equipped with an HP-5MS capillary column (30 m in length, 0.25 mm internal diameter, and 0.25 μm stationary phase film thick nesses). We followed the method as described by Yuan et al.^65^. The GC oven temperature was programmed at 60 °C for 1 min, then increased to 128 °C at a rate of 2.5 °C/min and maintained for 2 min, and at 5 °C/min to 250 °C (held for 6 min). High purity helium gas was used as mobile phase with a constant flow of 1 mL/min. The parameters for mass spectrometer were set as: 280 °C for both GC injector and MS transfer line, 300 °C for ion source. Mass spectra were recorded in the electron impact ionization (EI) at 70 eV. The MS scan range was from 50–550 m/z. The total ion chromatogram peak areas were used to calculate the percentages of the compositional compounds in essential oils. Chemical identification of each compositional compounds was based on their retention indices determined by reference to a homologous series of n-alkanes (C_5_-C_25:_Hc), and by comparisons of their mass spectral fragmentation patterns with those reported in the literature^66^, and their referenced mass spectra from the TSQ mass spectral library.

### Statistical analyses

Log-probit analysis (SPSS 24) was used to determine the median lethal concentration (LC_50_), the median knockdown concentration (KT_50_), regression equation, chi-square value and the 95% confidence limit. The numbers of mosquito landings and biting, on negative control, positive control and chemical-treated hands, were compared using an unpaired T-test. Further repellency data among treatments were analyzed using two-way ANOVA followed by Tukey’s honest significant difference (HSD) test (p < 0.05).

## Abbreviations

*Ar*: *Artemisia*
*An*: *Anopheles*
*Ar**. **argyi*: *Artemisia argyi*
*An**. **sinensis*: *Anopheles sinensis*
*Ae*: *Aedes*
CQ: Chongqing province
SC: Sichuan province
YN: Yunnan province
GS: Gansu province
HN: Henan province
HB: Hubei province
SD: Shandong province
